# Functional characterization of DLK1/MEG3 locus on chromosome 14q32.2 reveals the differentiation of pituitary neuroendocrine tumors

**DOI:** 10.18632/aging.202376

**Published:** 2020-12-29

**Authors:** Yiyuan Chen, Hua Gao, Qian Liu, Weiyan Xie, Bin Li, Sen Cheng, Jing Guo, Qiuyue Fang, Haibo Zhu, Zhuang Wang, Jichao Wang, Chuzhong Li, Yazhuo Zhang

**Affiliations:** 1 Department of Cell Biology, Beijing Neurosurgical Institute, Capital Medical University, Beijing 100070, China; 2 Department of Neurosurgery, Beijing Tiantan Hospital, Capital Medical University, Beijing 100070, China; 3 Department of Neurosurgery, The First Affiliated Hospital of Zhengzhou University, Zhengzhou 450052, China; 4 Department of Neurosurgery, People's Hospital of Xinjiang Uygur Autonomous Region, Xinjiang 830001, China; 5 China National Clinical Research Center for Neurological Diseases, Beijing 100070, China; 6 Brain Tumor Center, Beijing Institute for Brain Disorders, Beijing 100070, China

**Keywords:** pituitary neuroendocrine tumors, DLK1/MEG3 locus, somatotroph adenomas, differentiation, growth hormone secreting

## Abstract

Pituitary neuroendocrine tumors (PitNETs) represent the neoplastic proliferation of the anterior pituitary gland. Transcription factors play a key role in the differentiation of PitNETs. However, for a substantial proportion of PitNETs, the etiology is poorly understood. According to the transcription data of 172 patients, we found the imprinting disorders of the 14q32.2 region and DLK1/MEG3 locus associated with the differentiation of PitNETs. DLK1/MEG3 locus promoted somatotroph differentiation and inhibited tumor proliferation in PIT1(+) patients, furthermore, the level of DLK1 played a critical role in the trend of somatotroph or lactotroph differentiation. Anti-DLK1 monoclonal antibody blockade or siMEG3 both indicated that the DLK1/MEG3 significantly promoted the synthesis and secretion of GH/IGF-1 and inhibited cell proliferation. In addition, loss of DLK1 activated the mTOR signaling pathway in high DLK1-expressing and PIT1(+) GH3 cell lines, a mild effect in the low DLK1-expressing and PIT1(+) MMQ cell lines and no effect in the PIT1(-) ATT20 cell line. These findings emphasize that expression at the DLK1/MEG3 locus plays a key role in the differentiation of PitNETs, especially somatotroph adenomas, and provide potential molecular target data for patient stratification and treatment in the future.

## INTRODUCTION

Pituitary neuroendocrine tumors (PitNETs) represent the neoplastic proliferation of the anterior pituitary gland and account for 10-15% of intracranial tumors. Differentiation of pituitary cells mainly depends on transcription factors, including POU class 1 homeobox 1 (POU1F1, PIT1), T-box transcription factor 19 (TBX19, TPIT) and splicing factor 1 (SF1) [1–5]. As common functional PitNETs, somatotroph adenomas arise from PIT1 lineage cells and cause acromegaly due to excessive growth hormone (GH) and insulin-like growth factor 1 (IGF-1) levels. High serum GH/IGF-1 levels lead to comorbidities including arthritis, facial changes, prognathism, and glucose intolerance [6]. GH binds to two GH receptors (GHRs), activating a series of signaling molecules, which lead to changes in enzymatic activity, transport function, and gene expression that determine the final changes in growth and metabolism [7]. Surgery is the preferred treatment for somatotroph adenomas, and markers of remission include biochemical control, normal pituitary and parasellar MRI visualization, and recurrence-free postoperative duration [8–10]. Although tight medical control of GH/IGF-1 levels improves clinical outcomes, a significant number of patients exhibit persistent GH hypersecretion after treatment [11].

Genomic imprinting, a unique mechanism of epigenetic regulation that results in parent-of-origin-specific gene expression, is essential for normal mammalian development and growth [12]. In addition to microRNAs (miRNAs), long noncoding RNAs (lncRNAs) (>200 nt in length) have attracted attention due to their potential regulatory roles in biological processes [13]. Human chromosome 14q32.2 plays crucial roles in cell differentiation and tissue development, which includes an imprinted region containing paternally expressed genes (PEGs) encode delta like non-canonical Notch ligand 1 (DLK1), retrotransposon gag like 1 (RTL1), and iodothyronine deiodinase 3 (DIO3), maternally expressed genes (MEGs) encode MEG3/8/9, and several large clusters of miRNAs [14, 15]. The DLK1/MEG3 locus is mostly silenced in human nonfunctional PitNETs but not in functional PitNETs [16]. DLK1 is expressed throughout the developing pituitary gland and becomes restricted mostly to somatotroph cells within the adult gland; loss of DLK1 leads to a significant reduction in GH content throughout the lives of DLK1-null mice [17]. DLK1 is involved in PIT1-mediated transcription, and GH is a DLK1-regulated target gene in the GH3 cell line [18].

Increasing evidence has suggested alterations in gene expression as major contributors to the identification of disease-specific patterns in PitNETs, not infrequent recurrent somatic mutations [19, 20]. Through diversity analysis of the transcripts per million (TPM) values of the transcriptomic data and clinicopathological characteristics of 172 PitNETs, the imprinting disorders of the 14q32.2 region and DLK1/MEG3 locus associated with the differentiation of PitNETs were filtered. The DLK1/MEG3 locus functions in PitNETs, and its underlying mechanisms were largely assessed through anti-DLK1 monoclonal antibody blockade or siMEG3 in PitNET cell lines. Here, we showed the critical role of the DLK1/MEG3 locus in the differentiation of PitNETs in patients depending on the level of PIT1 and suggested the DLK1/MEG3 locus as a potential therapeutic target for somatotroph adenoma patients.

## RESULTS

### Clinical and pathological features of PitNETs

A total of 172 cohort samples were included for transcriptomic analysis; the samples were collected 92 males and 80 females with an average age of 49.65±0.92 years (20-75 years), and the average tumor volume was 12.94±1.61 cm^3^ (0.05-155.3 cm^3^) (Table 1). According to Knosp staging, Knosp stage III-IV samples were considered invasive (80 cases), and Knosp stage I-II samples were considered noninvasive (92 cases). Transsphenoidal surgery included initial surgery (135/172, 78.5%) and 37 cases of adenoma recurrence treated with surgery (37/172, 21.5%). Females were more susceptible to functional PitNETs than males (*P*<0.001).

**Table 1:** Clinic-pathological features of 172 PitNETs patients.

**Variable**	**Somatotroph**	**Lactotroph**	**Gonadotroph**	**Corticotroph**
Gender				
Male	15	6	62	9
Female	16	11	17	36
Age				
<30	3	4	2	3
30-39	6	1	9	8
40-49	11	4	24	16
50-59	7	5	26	9
≥60	4	3	18	9
Tumor volume				
(cm^3^)	8.35±1.65	14.54±3.76	12.09±2.59	16.66±3.71
Invasive				
Yes	12	11	29	28
No	19	6	50	17
Surgical extent				
Total resection	27	15	51	31
Partial resection	4	2	28	14
Operation				
Primary	21	17	67	31
Recurrence	10	0	12	15

### Identification of differentially expressed genes in PitNETs

**Figure 1:**
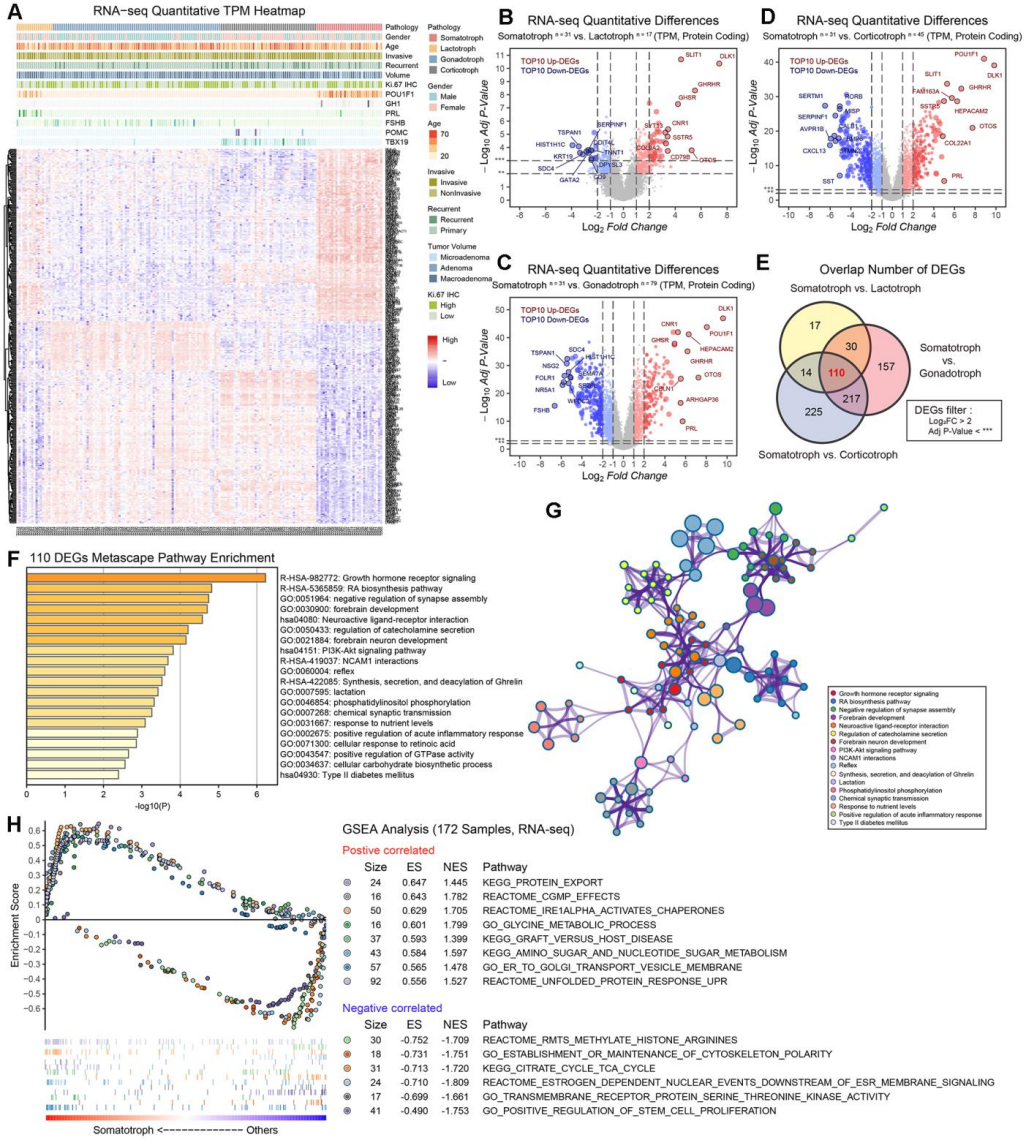
**The protein-coding RNA landscape in 172 PitNETs.** (**A**) Heatmap of unsupervised hierarchical clustering of the top 352 most variable genes among 31 somatotroph adenomas, 17 lactotroph adenomas, 79 gonadotroph adenomas and 45 corticotroph adenomas (|log_2_FC|>2, adj.*P*.val<0.001). Pathological and clinical annotations were provided. (**B**–**D**) Volcano plots showing the significantly differentially expressed genes among somatotroph adenomas vs. 3 other subtypes of PitNETs (up: red, down: blue), all log_2_FC of DLK1 > 7. (**E**) Venn diagram showing the intersection of DEGs among somatotroph adenomas vs. 3 other subtypes of PitNETs, DEGs were identified under the cutoff of adj.*P*.val < 0.001. (**F**, **G**) Metascape enrichment analysis including pathways and GO term of 110 overlapping DEGs in Figure 1E. (**H**) Outline of bioinformatic GSEA analysis.

In this study, we divided 172 patients into four groups according to the 2017 WHO classification of PitNETs for transcriptomic experiments; these groups included patients with somatotroph adenomas, lactotroph adenomas, corticotroph adenomas and gonadotroph adenomas. An unsupervised hierarchical clustering of the top 352 most variable genes revealed distinct gene expression profiles corresponding to the discretion of morpho-functional classification categories (Figure 1A). The volcano maps of the characteristic difference profiles of somatotroph adenomas are shown based on pairwise comparisons (Figure 1B–1D). A total of 110 molecules overlapped based on DEG filter parameters (|log_2_FC*|*>2, adjusted *P* value<0.001), and DLK1 (somatotroph vs. lactotroph: log_2_FC=7.397, somatotroph vs. gonadotroph: log_2_FC=9.611, somatotroph vs. corticotroph: log_2_FC=9.832) were the most significantly different in somatotroph adenomas compared with other subtypes (Figure 1E). Using the Metascape database, DEG pathway enrichment showed that the top three pathways were Growth hormone receptor signaling (R-HSA-982772), RA biosynthesis pathway (R-HSA-5365859) and Neuroactive ligand-receptor interaction (hsa04080), and the enriched GO terms focused on negative regulation of synapse assembly, forebrain development, and regulation of catecholamine secretion (Figure 1F, 1G). GSEA of 172 patients showed that the most positively correlated pathway was related to protein export and that the most negatively correlated pathway was related to methylated histone arginines in somatotroph adenomas compared with other subtypes (Figure 1H).

**Figure 2:**
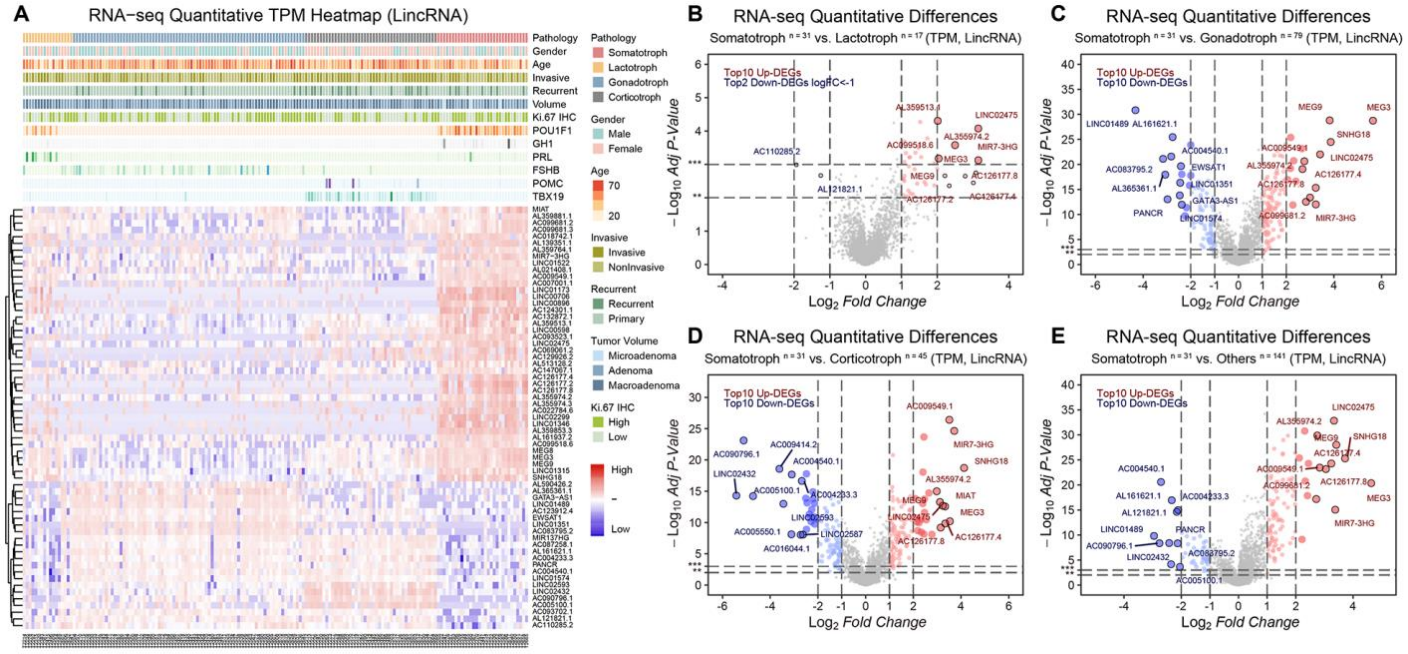
**The lncRNA landscape in 172 PitNETs.** (**A**) Heatmap of unsupervised hierarchical clustering of the top 63 most variable genes among 31 somatotroph adenomas, 17 lactotroph adenomas, 79 gonadotroph adenomas and 45 corticotroph adenomas (|log_2_FC|>2, adj.*P*.val<0.001). Pathological and clinical annotations were provided. (**B**–**D**) Volcano plots showing significantly differentially expressed genes somatotroph adenomas vs. 3 other subtypes of PitNETs (Up: red, Down: blue). (**E**) Volcano plots showing the AC126177.8, AC355974.2, LINC02475, MEG3, MEG9 and MiR7-3HG were the most significant lncRNAs, DEGs were identified by the R package limma, 31 somatotroph adenomas vs. 141 other subtype PitNETs.

LncRNAs that drive the transcriptome showed noticeable expression signatures in somatotroph adenomas compared with other subtypes of PitNETs. 41 upregulated lncRNAs and 22 downregulated lncRNAs in somatotroph adenomas were identified (|Log_2_FC|>2, adjusted *P* value<0.001): AC126177.8, AC355974.2, LINC02475, MEG3, MEG9 and MiR7-3HG were the most significantly upregulated DEGs (Figure 2A–2E).

### Description of the DLK1/MEG3 locus in 172 PitNETs

**Figure 3:**
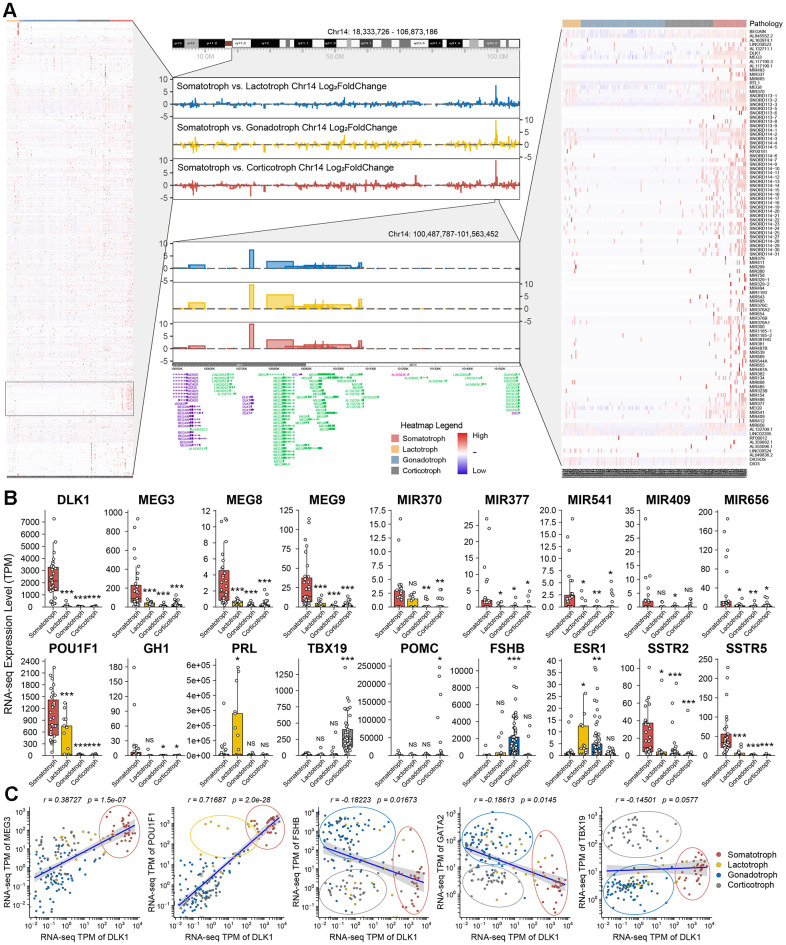
**Description of DLK1/MEG3 locus in 172 PitNETs.** (**A**) The imprinting disorders of 14q32.2 region was filtered based on genes distribution in Chromosome 14. A high-resolution profile showed the genomic positions and gene trees for DLK1/MEG3 locus. (**B**) The TPM values of DLK1/MEG3 and characteristic molecule in 172 PitNETs. (**C**) DLK1 combined transcription factors and immuophenotype could distinguish somatotroph, lactotroph, gonadotroph and corticotroph PitNET patients. *compare to control group *P*<0.05 **P<0.01 ***P<0.001.

The landscape of chromosome 14 was established in this study by transcriptomic experiments. The imprinting disorders of the 14q32.2 region in somatotroph adenomas compared with other subtypes were identified, and a high-resolution profile at 14q32.2 (chr14:100,487,787-101,563,452, including DLK1/MEG3 locus) is shown (Figure 3A). We observed that MEG3/8/9 and miR-377 in the 14q32.2 region showed a widespread increase in somatotroph adenomas (Figure 3B). The differentiation of PitNET cells depends on transcription factors, mainly POU1F1 (PIT1), TBX19 (TPIT), SF1, estrogen receptor 1 (ESR1, ER-α) and endothelial transcription factor GATA2 (GATA2). Intriguingly, we revealed distinct groups as follows: 1) somatotroph adenoma: high POU1F1 and high DLK1; 2) lactotroph adenoma: high POU1F1 and low DLK1; 3) gonadotroph adenoma: high FSHB and low DLK1, followed by high GATA2 and low DLK1; and 4) corticotroph adenoma: high TBX19 and low DLK1. In this study, SF1 or ESR1 did not show sensitivity or specificity for the classification of PitNETs (Figure 3C and Supplementary Figure 1).

### Clinical relevance of DLK1 in somatotroph adenoma

**Figure 4:**
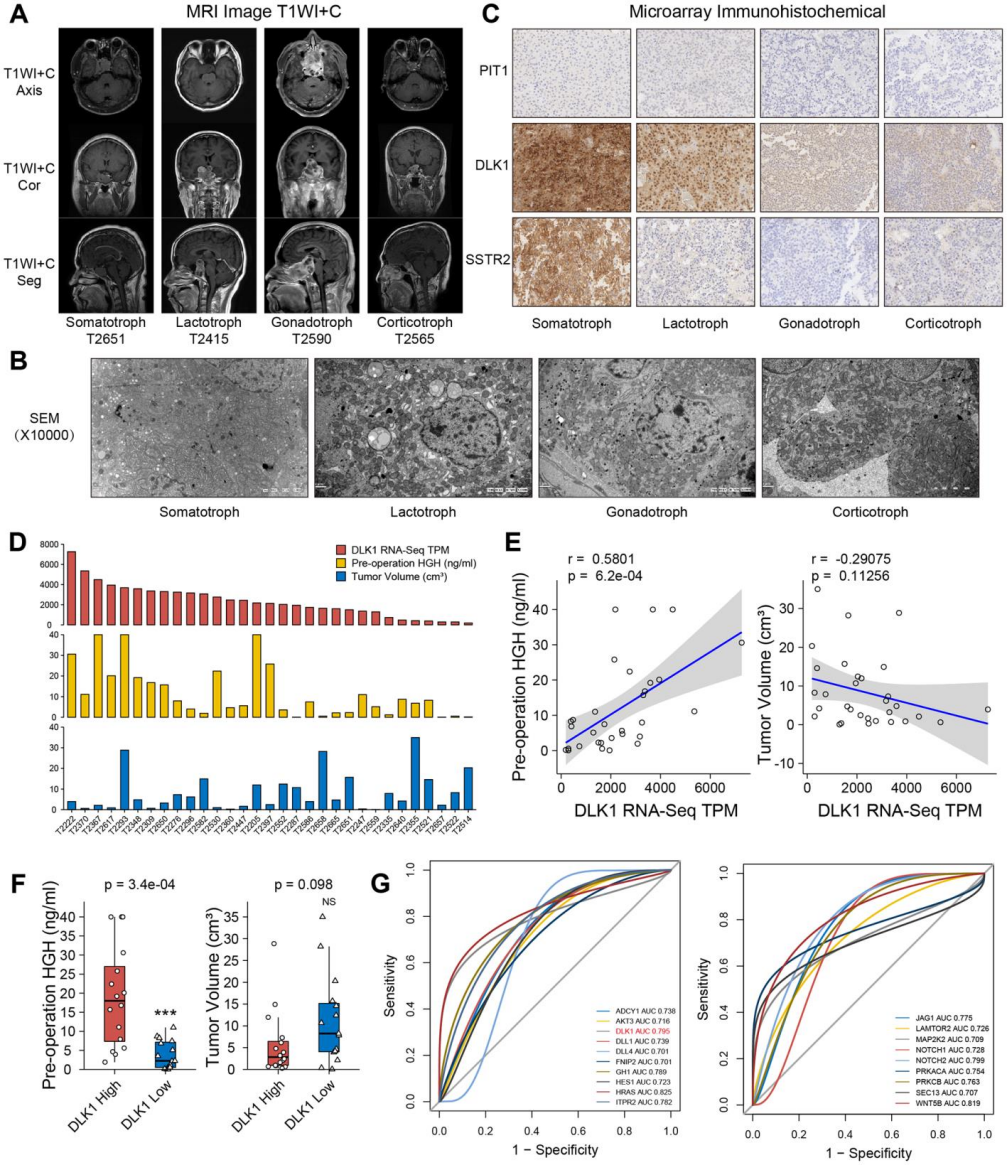
**Clinical relevance of DLK1 in somatotroph adenomas.** (**A**) MR images of PitNET patients. (**B**) Transmission electron microscope of PitNET patients. (**C**) Immunohistochemistry image of DLK1, PIT1 and SSTR2 in PitNET patients. 400×. (**D**) The TPM values of DLK1, serum GH levels and tumor volumes in 31 somatotroph adenomas. (**E**) Correlation of DLK1 and serum GH, tumor volume. (**F**) There was statistic difference of GH level between patients with high DLK1 and low DLK1, not tumor volume. (**G**) ROC curve. HRAS (0.825), WNT5B (0.819), Notch2 (0.799), DLK1 (0.795) and ITPR2 (0.782) were filtered in PI3K/AKT/mTOR pathway, Notch pathway and growth hormone synthesis, secretion and action. *compare to control group *P*<0.05 **P<0.01 ***P<0.001.

All patients were confirmed by MR image T1WI+C sequence and transmission electron microscopy (Figure 4A, 4B). To confirm the possibility of DLK1 can stratify PitNETs, we measured the levels of DLK1, PIT1 and Somatostatin receptor 2 (SSTR2) in these samples through immunohistochemistry staining (Figure 4C). The DLK1 expression levels yielded an H-score of 256.3±11.6 in somatotroph adenomas, 124.7±7.3 in lactotroph adenomas, 79±4.7 in gonadotroph adenomas, and 83.1±7.9 in corticotroph adenomas (*P*<0.001). The H-scores of PIT1 were 107.5±9.2, 102.4±8.9, 15.3±2.1, and 21.2±2.6, respectively (*P*<0.001). The H-scores of SSTR2 were 224.3±7.1, 31.5±1.7, 24.3±3.5, and 30.4±3.2, respectively (*P*<0.001).

In somatotroph adenomas from 31 transcriptome groups, patients with high DLK1 expression appeared to have higher preoperative serum GH levels than patients with low DLK1 expression (*r*=0.58, *P*<0.001). There was no significant difference in the correlation coefficient (*r*=-0.29, *P*=0.113) between the DLK1 level and tumor size (Figure 4D, 4E). We divided 31 patients into two groups based on the median DLK1 level: the high DLK1 group and the low DLK1 group. Patients in the high group had higher preoperative serum GH levels (*P*<0.001) and lower average tumor volumes (Figure 4F). Although there was no significant difference in tumor volume between the two groups, considering that individual extreme values do exist, we believe that they still have a negative correlation trend. Furthermore, we analyzed the potent risk factors related to the serum level of GH. Based on the area under the receiver operating characteristic (ROC) curve, we identified HRAS (0.825), WNT5B (0.819), Notch2 (0.799), DLK1 (0.795) and ITPR2 (0.782) in the PI3K/AKT/mTOR pathway, Notch pathway and growth hormone synthesis, secretion and action (Figure 4G).

### The effect of anti-DLK1 antibody on PitNET cell lines

**Figure 5:**
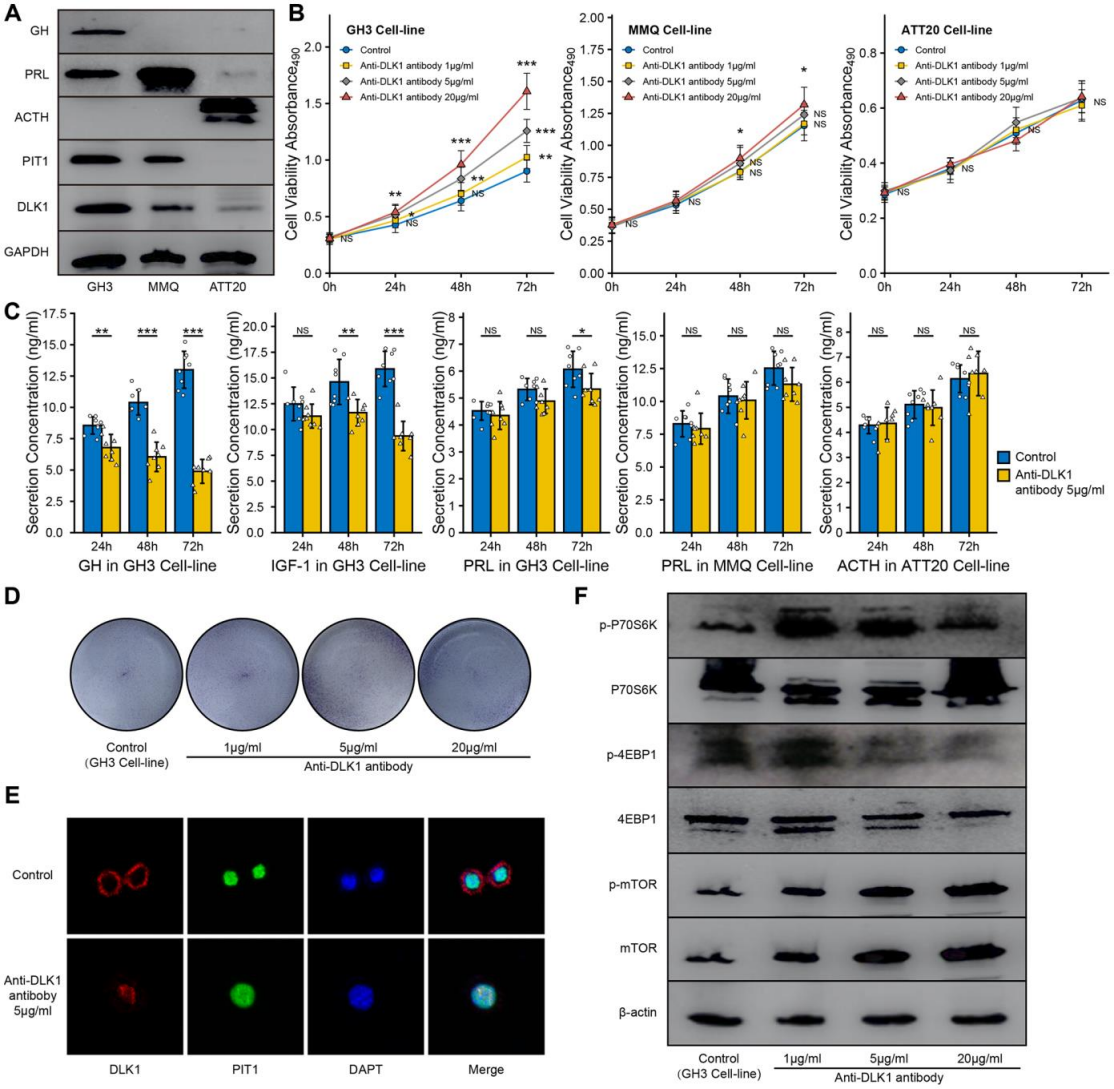
**Effect of anit-DLK1 antibody on the bioactivity of PitNET cell lines.** (**A**) Western blot assay measured the levels of DLK1 and PIT1 in GH3 cell line, MMQ cell line and ATT20 cell line. (**B**) Anti-DLK1 antibody inhibited the cell viability of GH3 cells in the dose- and time-dependent manner, not MMQ cells or ATT20 cells. (**C**) Anti-DLK1 antibody inhibited the secretion of GH/IGF-1 in GH3 cells, not PRL in MMQ cells and ACTH in ATT20 cells. (**D**) Clone forming experiment showed the anti-DLK1 antibody promoted the cell proliferation in GH3 cell line. (**E**) Confocal experiment showed DLK1 regulated the level of PIT1 in GH3 cell line. (**F**) Western blot experiment showed Anti-DLK1 antibody activated the mTOR pathway in GH3 cell line. *compare to control group *P*<0.05 **P<0.01 ***P<0.001.

In this study, we measured the levels of PIT1 and DLK1 in the GH3, MMQ and ATT20 cell lines. Western blot experiments showed that the GH3 and MMQ cell lines were PIT1(+) cell lines and that the ATT20 cell line was a PIT1(-) cell line (Figure 5A). Based on these results, according to the protein level, we defined GH3 cells as a high DLK1-expressing and PIT1(+) cell line and MMQ cells as a low DLK1-expressing and PIT1(+) cell line. We found that the anti-DLK-1 antibody could promote cell proliferation in a dose- and time-dependent manner in the GH3 cell line (*P*<0.05), and only a high concentration of the anti-DLK1 antibody (20 μg/ml) could promote cell proliferation in the MMQ cell line (*P*<0.05); there was no effect in the ATT20 cell line (Figure 5B). ELISA showed that the anti-DLK1 antibody caused the strongest inhibition of GH secretion (>70%), followed by IGF-1 secretion (almost 40%), and a slight inhibition of PRL secretion (Figure 5C). Clone forming experiments also suggested that the anti-DLK1 antibody promoted cell proliferation in the GH3 cell line (Figure 5D). Confocal images showed a decline in PIT1(green) accompanied by the blockade of DLK1 (red) in the GH3 cell line (Figure 5E). In addition, we found that the mTOR pathway was activated by the anti-DLK1 antibody in a dose-dependent manner in the GH3 cell line (Figure 5F).

### The effect of siMEG3 on PitNET cell lines

**Figure 6:**
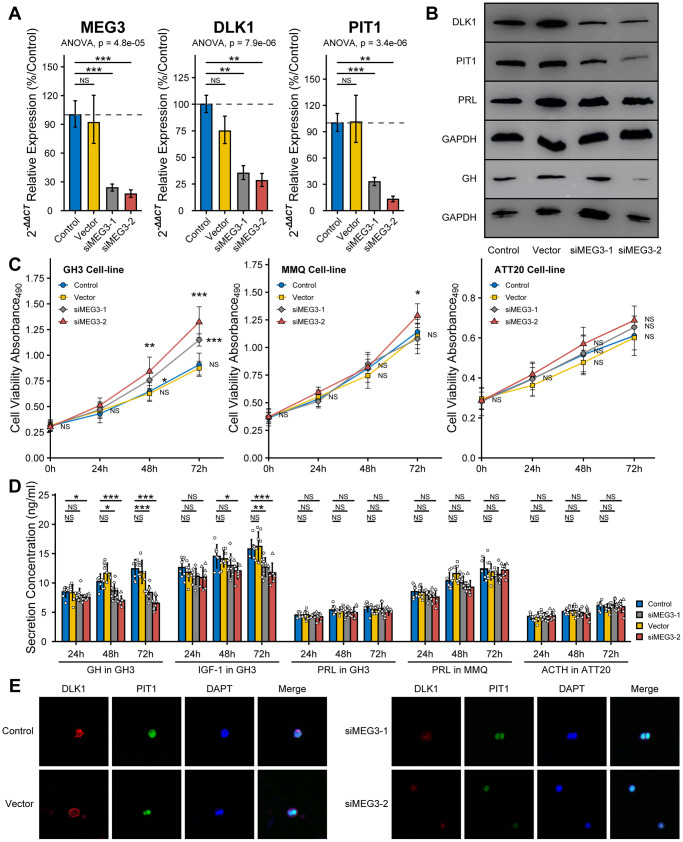
**Effect of siMEG3 on the bioactivity of PitNET cell lines.** (**A**) RT-qPCR experiment measured the RNAi efficiency of siMEG3-1 and siMEG3-2. (**B**) Western blot experiment showed RNAi-MEG3 reduced the level of DLK1, PIT1 and GH in GH3 cell line. (**C**) RNAi-MEG3 obviously increased the cell viability of GH3 cell line, mildly increase in MMQ cell line, and no change in ATT20 cell line. (**D**) RNAi-MEG3 inhibited the secretion of GH/IGF-1 in GH3 cells, not PRL in MMQ cells and ACTH in ATT20 cells. (**E**) Confocal experiment showed RNAi-MEG3 inhibited the levels of DLK1 and PIT1 in GH3 cells. *compare to control group *P*<0.05 **P<0.01 ***P<0.001.

To measure the effect of MEG3 on the bioactivity of PitNET cell lines, we synthesized fragments of small interfering RNA. The RT-qPCR assays proved that the RNAi efficiency of the siMEG3-1 and siMEG3-2 fragments in GH3 cells was more than 75% compared with the control or vector fragments. The RT-qPCR and western blot assays both showed inhibition of the expression levels of DLK1 and PIT1 after siMEG3 treatment (Figure 6A, 6B). Furthermore, the western blot assays showed that siMEG3 could inhibit the synthesis of GH but not of PRL in the GH3 cell line. siMEG3-1 and siMEG3-2 both promoted cell proliferation in the GH3 cell line after 48 h or 72 h of treatment (*P*<0.05), and only siMEG3-2 mildly promoted cell proliferation in the MMQ cell line after 72 h of treatment; however, no effect on the proliferation of the ATT20 cell line was observed, regardless of whether siMEG3-1 or siMEG3-2 was used (Figure 6C). ELISA also showed that siMEG3-1 and siMEG3-2 both inhibited the GH/IGF-1 levels in cell culture and had no effect on the secretion of PRL or ACTH by these cell lines (Figure 6D). Confocal assays showed a decrease in fluorescence intensity for DLK1 (red) and PIT1(green) after siMEG3-1 and siMEG3-2 treatment, especially siMEG3-2 (Figure 6E).

## DISCUSSION

Pituitary adenomas, which were recently renamed PitNETs, mostly represent benign neoplastic proliferation of the anterior pituitary gland. The 2017 WHO classification classifies PitNETs into seven morpho-functional types and three lineages. The French five-tiered classification emphasizes tumor proliferation and invasion, which provide prognostic value for the clinical treatment of PitNETs [5]. Mario et al. found gonadotroph signatures in some corticotroph and somatotroph PitNETs by integrated pangenomic analyses [4]. The low mutation burdens of PitNETs based on Whole genome sequencing (WGS) suggesting a limited impact of single nucleotide variations (SNVs) on the PitNET classifications [20–22]. In this study, we found that expression at the DLK1/MEG3 locus played a key role in the differentiation of PitNETs in patients depending on the level of PIT1, and we suggested that DLK1 may be used in addition to the current array of PitNET classifications.

Based on the DEGs of 172 PitNETs, DLK1 had the most obvious effect on distinguishing somatotroph adenomas from other subtype PitNETs with a high expression level over 2^7^ times. AC126177.8, AC355974.2, LINC02475, MEG3, MEG9 and MiR7-3HG were the most significantly different lncRNAs in somatotroph adenomas. We confirmed imprinting disorders in the 14q32.2 region by analyzing the landscape of chromosome 14. Combined with the clinical phenotype, DLK1 and MEG3 were identified as characteristic molecules in somatotroph adenomas. The transcriptomic and immunochemistry data also suggested that the expression level of DLK1 played a key role in the differentiation of PIT1(+) PitNETs. DLK1/MEG3 is an imprinted locus consisting of multiple maternally expressed lncRNA genes and PEGs [23, 24]. The expression dosage of DLK1 is functionally important, and modulation of its expression in a stem cell niche can profoundly alter the self-renewal properties of cells [25–27]. DLK1 plays important roles in the physiological adaptation associated with early life and is implicated in metabolic disease resistance; overexpression of DLK1 reduces fat stores, IGF-1 resistance, and defects in feedback regulation of GH in mice [28]. Ansell et al. reported GH as a target gene that is regulated by DLK1 through a PIT1-dependent mechanism [18]. These data supported our opinion that the level of DLK1 is the key factor for determining the differentiation of PIT1(+) PitNETs into somatotroph or lactotroph adenoma.

Traditionally, the DLK1/MEG3 locus plays a tumor suppressor role in human gonadotroph adenomas [16, 29]. Hypermethylation of IG-DMR at the DLK1/MEG3 locus is the reason for the loss of MEG3 that occurs only in gonadotroph adenomas, and no gene in the DLK1/MEG3 locus was significantly downregulated in somatotroph adenomas compared to other PitNETs [16, 30]. Overexpression of MEG3 could inhibit tumorigenesis and induce apoptosis in a pituitary-derived folliculostellate cell line [31]. It has been difficult to understand why somatotroph patients with higher serum GH levels have smaller tumor sizes and less invasive potential, while the DLK1 had the same trend of serum GH levels [32]. We speculated that the DLK1/MEG3 locus promoted somatotroph differentiation and inhibited tumor proliferation of PitNETs.

In this study, we measured the effect of DLK1/MEG3 on the bioactivity of the GH3 cell line (somatotroph), MMQ cell line (lactotroph) and ATT20 cell line (corticotroph). The western blot assays proved that the GH3 cell line was PIT1(+) with high DLK1 levels, the MMQ cell line was PIT1(+) with low DLK1 levels, and the ATT20 cell line was PIT1(-) with low DLK1 levels. Accompanied by cell proliferation, the blockade of the DLK1/MEG3 locus strongly inhibited the synthesis and secretion of GH/IGF in the GH3 cell line and mildly inhibited the synthesis and secretion of PRL, regardless of whether anti-DLK1 antibody or siMEG3 treatment was used. Blockade of the DLK1/MEG3 locus mildly increased cell proliferation but did not affect PRL secretion in the MMQ cell line. The change in the DLK1/MEG3 locus did not affect the bioactivity of the ATT20 cell line. Based on these results, we confirmed that DLK1/MEG3 promoted somatotroph differentiation and inhibited the tumorigenesis of PitNETs in PIT1-mediated transcription.

The DLK1/MEG3 locus suppresses the entire PI3K/AKT/mTOR pathway in hematopoietic stem cells by regulating mitochondrial biogenesis and metabolic activity [33]. We also noticed that DLK1 was negatively correlated with the citrate cycle in PitNET patients. This observation may explain the difference in the metabolic profile of somatotroph adenomas compared to other subtypes of patients. In our *in vitro* experiment, the anti-DLK1 antibody activated the mTOR pathway in GH3 cells.

**Figure 7:**
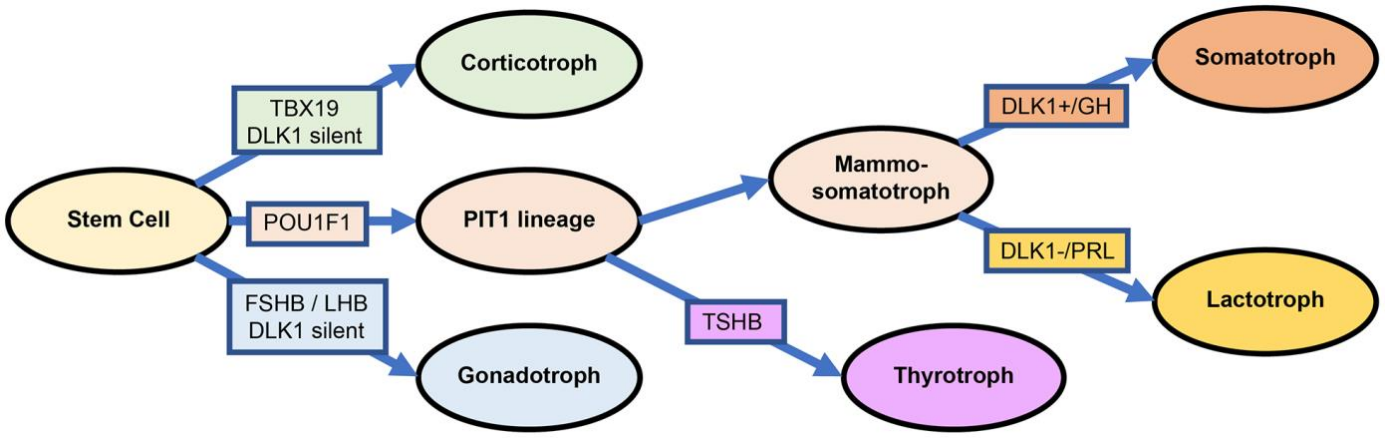
**Differentiation and transcription factors in PitNETs base on the DLK1 expression.**

In fact, the current 2017 WHO classification divides PitNETs into three transcription categories mainly based on the biological relevance of pituitary lineage factors, namely, PIT1, TPIT and SF1 [3]. However, the reliability of SF1 has been widely questioned due to variability across different research institutes, for example, in cytoplasmic and granular staining [34, 35]. There is no unique molecule that specifically distinguishes somatotroph adenoma from lactotroph adenoma, even for ER-α, as mentioned in the 2017 WHO classification criteria [2, 36]. Our lab reported that 27/50 somatotroph adenomas patients (54%) had high ER-α levels (definition: more than 50% positive cells), and only 5/42 lactotroph adenoma patients (11.9%) had high ER-α levels [37]. ER-α appeared to be more methylated in functioning corticotroph tumors than in functioning somatotroph adenomas (15 patients vs. 40 patients; *P*=0.025), which also provided evidence of high ER-α levels in somatotroph adenomas [38]. The evidence above provide that SF1 and ER-α are widespread in various subtypes of PitNETs. Based on the transcriptomic analysis of 172 PitNETs, we suggest a solution as follows: 1) somatotroph adenoma: high PIT1 and high DLK1; 2) lactotroph adenoma: high PIT1 and low DLK1; 3) gonadotroph adenoma: high FSHB and low PIT1/DLK1; and 4) corticotroph adenoma: high TBX19 and low PIT1/DLK1 (Figure 7). Certainly, more evidence should be provided for the exclusion of SF1 or ER-α in the morphological and functional classification of PitNETs.

This study presented an adjusted classification of PitNETs based on transcription factors. The DLK1/MEG3 imprint locus plays a critical role in somatotroph differentiation. The identification of biological mechanisms and potential clinical applications should lead to important improvements in anti-PitNET treatments. At least, combining analogs of somatostatin and blockade of the DLK1/MEG3 locus could be an effective treatment strategy for somatotroph adenomas, especially for patients with overactivation of the DLK1/MEG3 locus and resistance to standard treatment.

## MATERIALS AND METHODS

### Patient samples and cell lines

All the samples were obtained following transsphenoidal surgery performed at Beijing Tiantan Hospital from June 2018 to June 2019. Fresh tumor samples were stored in liquid nitrogen. 45 corticotroph adenomas, 79 gonadotroph adenomas, 17 lactotroph adenomas and 31 somatotroph adenomas from the study population (age range, 20–75 years) were diagnosed according to the 2017 World Health Organization classification of tumors of endocrine organs. The study protocols were approved by the Internal Review Board of Beijing Tiantan Hospital, which was affiliated with Capital Medical University, and conformed to the ethical guidelines of the Declaration of Helsinki (No. KY2016-035-01).

The GH3 and MMQ cell lines (ATCC, Manassas, VA, USA) cultured in a humidified incubator at 37° C and 5% CO_2_ in F-12K medium (ATCC) supplemented with 2.5% fetal bovine serum and 10% horse serum. The ATT20 cell line (ATCC) was cultured in a humidified incubator at 37° C and 5% CO_2_ in DMEM (ATCC) supplemented with 10% fetal bovine serum.

### RNA extractions, sequencing, transcriptomic data processing and analysis

For the RNA extractions, patient samples were processed with an AllPrep® DNA/RNA Mini kit (Qiagen, Hilden, Germany) according to the manufacturer’s instructions. The quantity and quality of the RNA was evaluated by an RNA Nano6000 assay kit (Aligent Technologies, CA, USA) (RIN>6.8). RNA (3 μg/sample) was used for the RNA preparations, and the ribosomal RNA was removed using an Epicentre Ribo-zero™ rRNA Removal Kit (Epicentre, Madison, WI, USA). The sequencing library was generated using the NEBNext® Ultra™ Directional RNA Library Prep Kit (NEB, Ispawich, USA). The library fragments (150~200 bp) were purified by the AMPure XP system (Beckman Coulter, Beverly, USA) and then assessed by the Agilent Bioanalyzer 2100 system. The libraries were sequenced on an Illumina HiSeq X platform, and then, 150-bp paired-end reads were generated. Reads containing adapters, reads containing poly-N and low-quality reads were removed. The paired-end clean reads were aligned to the human reference genome (hg19) using Hisat2 (v2.0.5) [39]. HTSeq (v0.11.2) was used to count the read numbers mapped to each gene [40]. The R package limma was used to analyze the quantitative differentiation between two identified groups [41]. KEGG pathway enrichment and GO term results were exported from the Metascape website with filtered differential gene data input [42]. The R package ClusterProfiler was used to process the GSEA analysis [43].

### Tissue microarray construction and immunochemistry staining

The formalin-fixed, paraffin-embedded tissue blocks were sectioned. Three core biopsies (2.0 mm in diameter) were selected from the paraffin-embedded tissue. The cores were transferred to tissue microarrays using a semiautomated system (Aphelys MiniCore, Mitogen, UK). The microarrays were cut into 4-μm sections and incubated with anti-DLK1 (rabbit monoclonal, 1:600, ab210471, Abcam), anti-PIT1(mouse monoclonal, 1:500, sc393943, Santa Cruz) and anti-SSTR2 (rabbit monoclonal, 1:400, ab134152, Abcam) primary antibodies. BondTM Ploymer Refine Detection (Leica Biosystems, DS9800) was used for the detection of the primary antibodies. The slides were scanned into digital pictures, and expression was examined using Aperio AT2 (Leica Biosystems). The H-score was obtained by multiplying the staining intensity by a constant to adjust the mean to the strongest intensity [H-score = 3×(percentage of strong staining)] (1.0%, weak; 2.0%, moderate; 3.0%, strong) to yield a score ranging from 0 to 300.

### Cell proliferation and colony formation assay

The number of cells in suspension was adjusted to 1×10^5^/ml, and 100 μl cell suspension was plated into each well of a 96-well plate. After overnight incubation, anti-DLK1 monoclonal antibody blockade (1 μg/ml, 5 μg/ml, and 20 μg/ml) or siMEG3 fragments (Supplementary Table 1) were added.

The cells were then cultured for 24 h, 48 h and 72 h; 20 μl 3-(4,5-diethylthiazol-2-yl)-5-(3-carboxymethoxyphenyl)-2-(4-sulfophenyl)-2H-tetrazolium, inner salt (MTS) solution was then added into each well and incubated for an additional 4 h. The absorbance at 490 nm was measured with an ELISA plate reader (Thermo, USA). For the colony formation assay, a total of 200 cells were plated into each well of a 24-well plate and maintained for 7 days. The colonies were then fixed with 4% formaldehyde for 30 min and stained with 0.2% crystal violet for 10 min. Triplicate wells were measured in each treatment group.

### Immunofluorescence and confocal microscopy

For the immunofluorescence experiments, GH3 cells were seeded on poly-L-lysine-coated confocal dishes, and an anti-DLK1 antibody (5 μg/ml) was added. After 24 h in culture, the cells were fixed with 4% paraformaldehyde for 30 min, washed with PBS, and permeabilized with 0.2% Triton X-100 in PBS for 15 min at room temperature. The fixed cells were incubated with an anti-DLK1 (1:600) antibody and anti-PIT1 antibody (1:500) overnight at 4° C followed by incubation with the fluorescently (Alexa Fluor 488 and 594) labeled secondary antibody (1:300) for 1 h at room temperature. The cell nuclei were visualized by DAPI counterstaining (Invitrogen).

### ELISA assay

The adrenocorticotropic hormone (ACTH), GH, IGF-1, and prolactin (PRL) levels in 10 μl cell culture supernatant were detected by ELISA kits (APPLYGEN) according to the protocol. The absorbance of each well at 450 nm was measured using an ELISA plate reader (Thermo Fisher).

### Reverse transcription and quantitative PCR

Microarray hybridization and RT-qPCR were performed as previously described [44]. The total RNA of 30 samples was extracted and purified using the Rneasy®Mini Kit (QiaGen, Hilden, Germany) following the manufacturer’s instructions. RT-qPCR was performed on a QuantStudio5 (Applied Biosystems, Singapore) with primers of each genes (Supplementary Table 2). The fold-change in the differential expression of each gene was calculated using the comparative CT method (2−ΔΔCT method) in the R package PCR, with GAPDH as the reference gene [45].

### SDS-PAGE and western blot analyses

Samples (up to 10 mg) were lysed in lysis buffer containing 1% Nonidet P-40 (Calbiochem, Merck, Darmstadt, Germany) and protease and phosphatase inhibitor cocktails (Roche, IL, USA) overnight at 4° C. The total extracts were centrifuged at 12,000 g for 30 min at 4° C, and the protein concentration was determined by the BCA method (Pierce Biotechnology, IL, USA). A total of 20 μg of protein per lane was loaded onto 10% Bis-Tris SDS-PAGE gels, separated electrophoretically, and blotted onto polyvinylidene fluoride membranes (Merck). The blots were incubated with anti-ACTH (rabbit polyclonal, 1:2,000, AB74976), anti-GH (mouse monoclonal, 1:1,000, ab155276), anti-PRL (rabbit polyclonal, 1:1,000, ab64377), anti-DLK1 (1:2,000, Abcam), anti-PIT1(1:2,000, Santa-Cruz), anti-phospho-4EBP1 (rabbit polyclonal, 1:2,000, AF3432, Affbiotech), anti-4EBP1 antibody (mouse monoclonal, 1:1,000, 60246-1, Proteintech), anti-phospho-P70S6K (rabbit polyclonal, 1:1,000, AF3228, Affbiotech), anti-P70S6K antibody (rabbit polyclonal, 1:1,000, 144851-AP, Proteintech), anti-phospho-mTOR-2448 (rabbit monoclonal, 5536s, 1:1,000, CST) and anti-mTOR (rabbit monoclonal, 1:1,000, 2983s, CST) antibodies, followed by a secondary antibody (1:8,000) tagged with horseradish peroxidase (Santa Cruz Biotechnology). The blots were visualized by enhanced chemiluminescence, and densitometry was performed using a fluorescence image analyzer (Amersham Imager 600, GE, MA, USA). GAPDH was used as the loading control.

### Statistical analysis

All the statistical analyses were conducted using SPSS Statistics Version 22 (IBM Corporation, Armonk, New York, USA). Unpaired Student’s test and chi-square (Fisher’s exact) test were used to compare quantitative and qualitative data. The *P* value of less than 0.05 was considered significant. The R package pROC was used to compute ROC curves, AUC, and *P* values to evaluate the predictive accuracy of selected genes [46].

### Ethics approval and consent to participate

The study protocols were approved by the Internal Review Board of Beijing Tiantan Hospital, which was affiliated to Capital Medical University, and conformed to the ethical guidelines of the Declaration of Helsinki (No. KY2016-035-01).

### Availability of data and materials

All the data generated or analyzed in this study are included in this published article and its Additional files.

## Supplementary Material

Supplementary Figure 1

Supplementary Tables

## References

[1] Molitch ME. Diagnosis and treatment of pituitary adenomas: a review. JAMA. 2017; 317:516–24. 10.1001/jama.2016.19699 28170483

[2] Mete O, Lopes MB. Overview of the 2017 WHO classification of pituitary tumors. Endocr Pathol. 2017; 28:228–43. 10.1007/s12022-017-9498-z 28766057

[3] Lloyd R, Osamura R, Klöppel G, Rosai J. (2017) WHO classification of tumours of the endocrine organs, 4th edn. Lyon: International Agency for Research on Cancer;

[4] Neou M, Villa C, Armignacco R, Jouinot A, Raffin-Sanson ML, Septier A, Letourneur F, Diry S, Diedisheim M, Izac B, Gaspar C, Perlemoine K, Verjus V, et al. Pangenomic classification of pituitary neuroendocrine tumors. Cancer Cell. 2020; 37:123–34.e5. 10.1016/j.ccell.2019.11.002 31883967

[5] Trouillas J, Jaffrain-Rea ML, Vasiljevic A, Raverot G, Roncaroli F, Villa C. How to classify the pituitary neuroendocrine tumors (PitNET)s in 2020. Cancers (Basel). 2020; 12:514. 10.3390/cancers12020514 32098443 PMC7072139

[6] Melmed S. Acromegaly pathogenesis and treatment. J Clin Invest. 2009; 119:3189–202. 10.1172/JCI39375 19884662 PMC2769196

[7] Cirillo F, Lazzeroni P, Sartori C, Street ME. Inflammatory diseases and growth: effects on the GH-IGF axis and on growth plate. Int J Mol Sci. 2017; 18:1878. 10.3390/ijms18091878 28858208 PMC5618527

[8] Ezzat S, Caspar-Bell GM, Chik CL, Denis MC, Domingue MÈ, Imran SA, Johnson MD, Lochnan HA, Grégoire Nyomba BL, Prebtani A, Ridout R, Ramirez JA, Van Uum S. Predictive markers for postsurgical medical management of acromegaly: a systematic review and consensus treatment guideline. Endocr Pract. 2019; 25:379–93. 10.4158/EP-2018-0500 30657362

[9] Racine MS, Barkan AL. Medical management of growth hormone-secreting pituitary adenomas. Pituitary. 2002; 5:67–76. 10.1023/a:1022356313153 12675503

[10] Mehta GU, Lonser RR. Management of hormone-secreting pituitary adenomas. Neuro Oncol. 2017; 19:762–73. 10.1093/neuonc/now130 27543627 PMC5464431

[11] Melmed S. Pituitary-tumor endocrinopathies. N Engl J Med. 2020; 382:937–50. 10.1056/NEJMra1810772 32130815

[12] Ferguson-Smith AC. Genomic imprinting: the emergence of an epigenetic paradigm. Nat Rev Genet. 2011; 12:565–75. 10.1038/nrg3032 21765458

[13] Luo M, Jeong M, Sun D, Park HJ, Rodriguez BA, Xia Z, Yang L, Zhang X, Sheng K, Darlington GJ, Li W, Goodell MA. Long non-coding RNAs control hematopoietic stem cell function. Cell Stem Cell. 2015; 16:426–38. 10.1016/j.stem.2015.02.002 25772072 PMC4388783

[14] Kagami M, Sekita Y, Nishimura G, Irie M, Kato F, Okada M, Yamamori S, Kishimoto H, Nakayama M, Tanaka Y, Matsuoka K, Takahashi T, Noguchi M, et al. Deletions and epimutations affecting the human 14q32.2 imprinted region in individuals with paternal and maternal upd(14)-like phenotypes. Nat Genet. 2008; 40:237–42. 10.1038/ng.2007.56 18176563

[15] Howard M, Charalambous M. Molecular basis of imprinting disorders affecting chromosome 14: lessons from murine models. Reproduction. 2015; 149:R237–49. 10.1530/REP-14-0660 25820903

[16] Cheunsuchon P, Zhou Y, Zhang X, Lee H, Chen W, Nakayama Y, Rice KA, Tessa Hedley-Whyte E, Swearingen B, Klibanski A. Silencing of the imprinted DLK1-MEG3 locus in human clinically nonfunctioning pituitary adenomas. Am J Pathol. 2011; 179:2120–30. 10.1016/j.ajpath.2011.07.002 21871428 PMC3181372

[17] Cheung LY, Rizzoti K, Lovell-Badge R, Le Tissier PR. Pituitary phenotypes of mice lacking the notch signalling ligand delta-like 1 homologue. J Neuroendocrinol. 2013; 25:391–401. 10.1111/jne.12010 23279263 PMC3664429

[18] Ansell PJ, Zhou Y, Schjeide BM, Kerner A, Zhao J, Zhang X, Klibanski A. Regulation of growth hormone expression by delta-like protein 1 (Dlk1). Mol Cell Endocrinol. 2007; 271:55–63. 10.1016/j.mce.2007.04.002 17485162 PMC1974851

[19] Song ZJ, Reitman ZJ, Ma ZY, Chen JH, Zhang QL, Shou XF, Huang CX, Wang YF, Li SQ, Mao Y, Zhou LF, Lian BF, Yan H, et al. The genome-wide mutational landscape of pituitary adenomas. Cell Res. 2016; 26:1255–59. 10.1038/cr.2016.114 27670697 PMC5099864

[20] Salomon MP, Wang X, Marzese DM, Hsu SC, Nelson N, Zhang X, Matsuba C, Takasumi Y, Ballesteros-Merino C, Fox BA, Barkhoudarian G, Kelly DF, Hoon DS. The epigenomic landscape of pituitary adenomas reveals specific alterations and differentiates among acromegaly, Cushing’s disease and endocrine-inactive subtypes. Clin Cancer Res. 2018; 24:4126–36. 10.1158/1078-0432.CCR-17-2206 30084836

[21] Reincke M, Sbiera S, Hayakawa A, Theodoropoulou M, Osswald A, Beuschlein F, Meitinger T, Mizuno-Yamasaki E, Kawaguchi K, Saeki Y, Tanaka K, Wieland T, Graf E, et al. Mutations in the deubiquitinase gene USP8 cause Cushing’s disease. Nat Genet. 2015; 47:31–38. 10.1038/ng.3166 25485838

[22] Li C, Xie W, Rosenblum JS, Zhou J, Guo J, Miao Y, Shen Y, Wang H, Gong L, Li M, Zhao S, Cheng S, Zhu H, et al. Somatic SF3B1 hotspot mutation in prolactinomas. Nat Commun. 2020; 11:2506. 10.1038/s41467-020-16052-8 32427851 PMC7237453

[23] Traustadóttir GÁ, Lagoni LV, Ankerstjerne LB, Bisgaard HC, Jensen CH, Andersen DC. The imprinted gene delta like non-canonical notch ligand 1 (Dlk1) is conserved in mammals, and serves a growth modulatory role during tissue development and regeneration through notch dependent and independent mechanisms. Cytokine Growth Factor Rev. 2019; 46:17–27. 10.1016/j.cytogfr.2019.03.006 30930082

[24] Abi Habib W, Brioude F, Azzi S, Rossignol S, Linglart A, Sobrier ML, Giabicani É, Steunou V, Harbison MD, Le Bouc Y, Netchine I. Transcriptional profiling at the DLK1/MEG3 domain explains clinical overlap between imprinting disorders. Sci Adv. 2019; 5:eaau9425. 10.1126/sciadv.aau9425 30801013 PMC6382400

[25] da Rocha ST, Charalambous M, Lin SP, Gutteridge I, Ito Y, Gray D, Dean W, Ferguson-Smith AC. Gene dosage effects of the imprinted delta-like homologue 1 (Dlk1/Pref1) in development: implications for the evolution of imprinting. PLoS Genet. 2009; 5:e1000392. 10.1371/journal.pgen.1000392 19247431 PMC2640098

[26] Ferrón SR, Charalambous M, Radford E, McEwen K, Wildner H, Hind E, Morante-Redolat JM, Laborda J, Guillemot F, Bauer SR, Fariñas I, Ferguson-Smith AC. Postnatal loss of Dlk1 imprinting in stem cells and niche astrocytes regulates neurogenesis. Nature. 2011; 475:381–85. 10.1038/nature10229 21776083 PMC3160481

[27] Cleaton MA, Dent CL, Howard M, Corish JA, Gutteridge I, Sovio U, Gaccioli F, Takahashi N, Bauer SR, Charnock-Jones DS, Powell TL, Smith GC, Ferguson-Smith AC, Charalambous M. Fetus-derived DLK1 is required for maternal metabolic adaptations to pregnancy and is associated with fetal growth restriction. Nat Genet. 2016; 48:1473–80. 10.1038/ng.3699 27776119 PMC5373434

[28] Charalambous M, Da Rocha ST, Radford EJ, Medina-Gomez G, Curran S, Pinnock SB, Ferrón SR, Vidal-Puig A, Ferguson-Smith AC. DLK1/PREF1 regulates nutrient metabolism and protects from steatosis. Proc Natl Acad Sci USA. 2014; 111:16088–93. 10.1073/pnas.1406119111 25349437 PMC4234615

[29] Butz H, Likó I, Czirják S, Igaz P, Korbonits M, Rácz K, Patócs A. MicroRNA profile indicates downregulation of the TGFβ pathway in sporadic non-functioning pituitary adenomas. Pituitary. 2011; 14:112–24. 10.1007/s11102-010-0268-x 21063788

[30] Gejman R, Batista DL, Zhong Y, Zhou Y, Zhang X, Swearingen B, Stratakis CA, Hedley-Whyte ET, Klibanski A. Selective loss of MEG3 expression and intergenic differentially methylated region hypermethylation in the MEG3/DLK1 locus in human clinically nonfunctioning pituitary adenomas. J Clin Endocrinol Metab. 2008; 93:4119–25. 10.1210/jc.2007-2633 18628527 PMC2579639

[31] Zhu D, Xiao Z, Wang Z, Hu B, Duan C, Zhu Z, Gao N, Zhu Y, Wang H. MEG3/MIR-376B-3P/HMGA2 axis is involved in pituitary tumor invasiveness. J Neurosurg. 2020. [Epub ahead of print]. 10.3171/2019.10.JNS191959 31899875

[32] Hauser BM, Lau A, Gupta S, Bi WL, Dunn IF. The epigenomics of pituitary adenoma. Front Endocrinol (Lausanne). 2019; 10:290. 10.3389/fendo.2019.00290 31139150 PMC6527758

[33] Qian P, He XC, Paulson A, Li Z, Tao F, Perry JM, Guo F, Zhao M, Zhi L, Venkatraman A, Haug JS, Parmely T, Li H, et al. The Dlk1-Gtl2 locus preserves LT-HSC function by inhibiting the PI3K-mTOR pathway to restrict mitochondrial metabolism. Cell Stem Cell. 2016; 18:214–28. 10.1016/j.stem.2015.11.001 26627594 PMC5545934

[34] McDonald WC, Banerji N, McDonald KN, Ho B, Macias V, Kajdacsy-Balla A. Steroidogenic factor 1, Pit-1, and adrenocorticotropic hormone: a rational starting place for the immunohistochemical characterization of pituitary adenoma. Arch Pathol Lab Med. 2017; 141:104–12. 10.5858/arpa.2016-0082-OA 27227698

[35] Manojlovic-Gacic E, Engström BE, Casar-Borota O. Histopathological classification of non-functioning pituitary neuroendocrine tumors. Pituitary. 2018; 21:119–29. 10.1007/s11102-017-0855-1 29275530 PMC5849671

[36] Lopes MB. The 2017 world health organization classification of tumors of the pituitary gland: a summary. Acta Neuropathol. 2017; 134:521–35. 10.1007/s00401-017-1769-8 28821944

[37] Gao H, Xue Y, Cao L, Liu Q, Liu C, Shan X, Wang H, Gu Y, Zhang Y. ESR1 and its antagonist fulvestrant in pituitary adenomas. Mol Cell Endocrinol. 2017; 443:32–41. 10.1016/j.mce.2016.12.029 28043824

[38] García-Martínez A, Sottile J, Sánchez-Tejada L, Fajardo C, Cámara R, Lamas C, Barberá VM, Picó A. DNA methylation of tumor suppressor genes in pituitary neuroendocrine tumors. J Clin Endocrinol Metab. 2019; 104:1272–82. 10.1210/jc.2018-01856 30423170

[39] Baruzzo G, Hayer KE, Kim EJ, Di Camillo B, FitzGerald GA, Grant GR. Simulation-based comprehensive benchmarking of RNA-seq aligners. Nat Methods. 2017; 14:135–39. 10.1038/nmeth.4106 27941783 PMC5792058

[40] Anders S, Pyl PT, Huber W. HTSeq—a python framework to work with high-throughput sequencing data. Bioinformatics. 2015; 31:166–69. 10.1093/bioinformatics/btu638 25260700 PMC4287950

[41] Ritchie ME, Phipson B, Wu D, Hu Y, Law CW, Shi W, Smyth GK. Limma powers differential expression analyses for RNA-sequencing and microarray studies. Nucleic Acids Res. 2015; 43:e47. 10.1093/nar/gkv007 25605792 PMC4402510

[42] Zhou Y, Zhou B, Pache L, Chang M, Khodabakhshi AH, Tanaseichuk O, Benner C, Chanda SK. Metascape provides a biologist-oriented resource for the analysis of systems-level datasets. Nat Commun. 2019; 10:1523. 10.1038/s41467-019-09234-6 30944313 PMC6447622

[43] Yu G, Wang LG, Han Y, He QY. clusterProfiler: an R package for comparing biological themes among gene clusters. OMICS. 2012; 16:284–87. 10.1089/omi.2011.0118 22455463 PMC3339379

[44] Feng J, Wang J, Liu Q, Li J, Zhang Q, Zhuang Z, Yao X, Liu C, Li Y, Cao L, Li C, Gong L, Li D, et al. DAPT, a γ-secretase inhibitor, suppresses tumorigenesis, and progression of growth hormone-producing adenomas by targeting notch signaling. Front Oncol. 2019; 9:809. 10.3389/fonc.2019.00809 31508369 PMC6718711

[45] Pabinger S, Rödiger S, Kriegner A, Vierlinger K, Weinhäusel A. A survey of tools for the analysis of quantitative PCR (qPCR) data. Biomol Detect Quantif. 2014; 1:23–33. 10.1016/j.bdq.2014.08.002 27920994 PMC5129434

[46] Robin X, Turck N, Hainard A, Tiberti N, Lisacek F, Sanchez JC, Müller M. pROC: an open-source package for R and S+ to analyze and compare ROC curves. BMC Bioinformatics. 2011; 12:77. 10.1186/1471-2105-12-77 21414208 PMC3068975

