# Supplementary material for: Functional characterization of DLK1/MEG3 locus on chromosome 14q32.2 reveals the differentiation of pituitary neuroendocrine tumors
Source: Aging (Albany NY). 2020 Dec 29;13(1):1422–39. doi: 10.18632/aging.202376 (PMC7835058; doi:10.18632/aging.202376)
Supplement: Supplementary Figure 1 [file aging-13-202376-s001.pdf]

SUPPLEMENTARY FIGURE

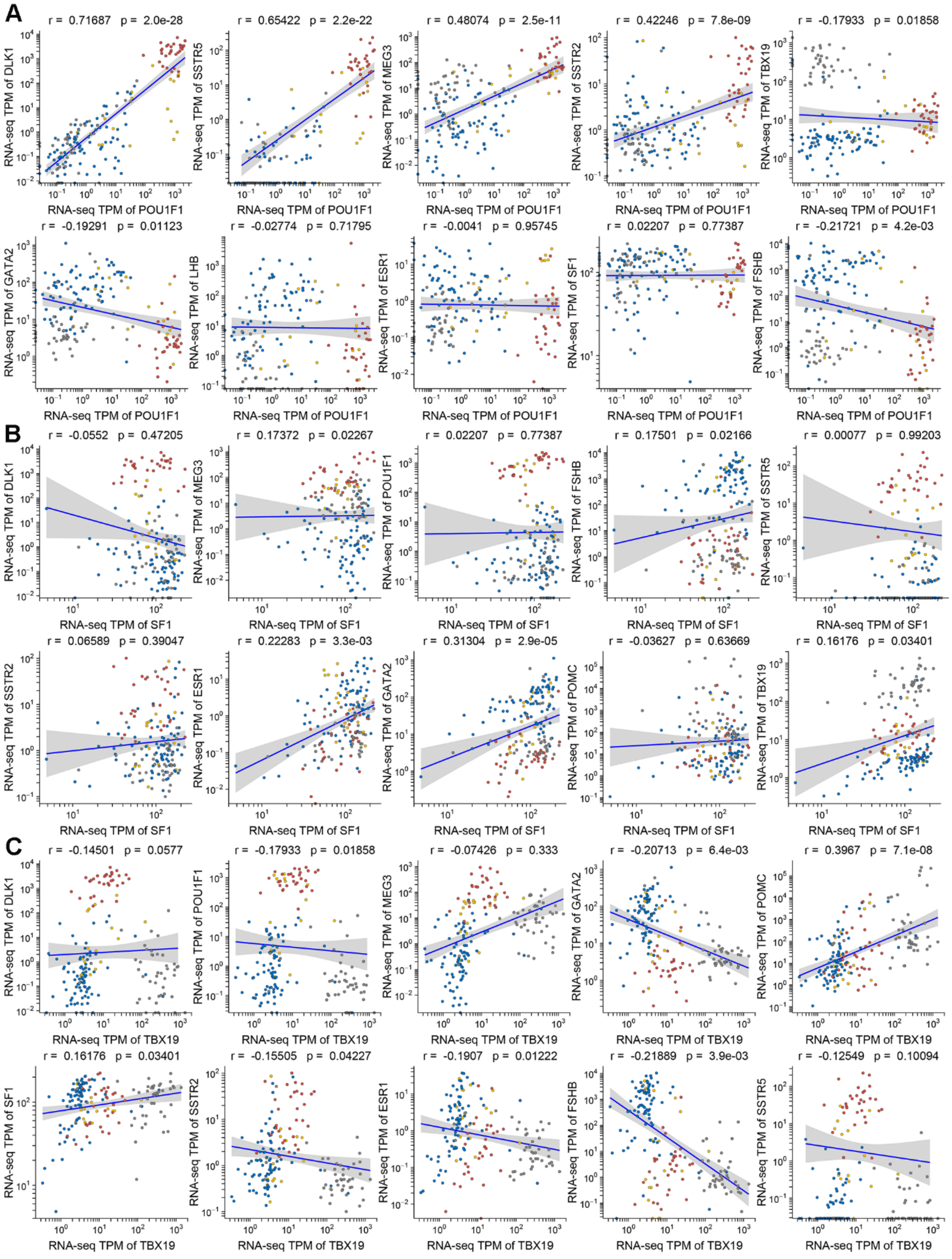

Supplementary Figure 1. DLK1, POU1F1, TBX19 can distinguish somatotroph, lactotroph, gonadotroph, and corticotroph PitNETs distinctly and form the subtypes to aggregation clusters. (**A**) The clustering effects with the base of POU1F1 and DLK1 can distinguish the subtypes of PitNETs, especially the somatotroph subtype. (**B**) The clustering effects with the base of SF1 and ESR1 are not clear enough to distinguish the subtypes of PitNETs. (**C**) The clustering effects with the base of TBX19 and DLK1 can distinguish the subtypes of PitNETs, especially the gonadotroph and corticotroph subtypes. Red: somatotroph; Yellow: lactotroph; Blue: gonadotroph; Gray: corticotroph.
