# Supplementary material for: Functional characterization of DLK1/MEG3 locus on chromosome 14q32.2 reveals the differentiation of pituitary neuroendocrine tumors
Source: Aging (Albany NY). 2020 Dec 29;13(1):1422–39. doi: 10.18632/aging.202376 (PMC7835058; doi:10.18632/aging.202376)
Supplement: Supplementary Tables [file aging-13-202376-s002.pdf]

SUPPLEMENTARY TABLES

Supplementary Table 1. Fragment in small RNA interference experiment.

Fragment	Sequence
siMEG3-1	
forward	GGAAUGAGCAUGUGGCUGATT
reverse	UCAGCCACAUGCUCAUUCCTT
siMEG3-2	
forward	CCACUAGCAUACAGAACAATT
reverse	UUGUUCUGUAUGCUAGUGGTT

Supplementary Table 2. Primers in RT-qPCR experiment.

Primer	Sequence
MEG3	
forward	CGAACGGCTCTTGCTCAGGTT
reverse	GCACAACAGGAAATGGCACAGG
DLK1	
forward	AAGAAGAAGAACCTGCTGTT
reverse	GGACACGCTGCTTAGATAT
PIT1	
forward	GTCAAACAACCATCTGCCGA
reverse	CTGCGAAGAAGGTTTGCTGT
